# Synergistic Mutations of *LRP6* and *WNT10A* in Familial Tooth Agenesis

**DOI:** 10.3390/jpm11111217

**Published:** 2021-11-17

**Authors:** Kuan-Yu Chu, Yin-Lin Wang, Yu-Ren Chou, Jung-Tsu Chen, Yi-Ping Wang, James P. Simmer, Jan C.-C. Hu, Shih-Kai Wang

**Affiliations:** 1 Department of Dentistry, School of Dentistry, National Taiwan University, Taipei City 100, Taiwan; b93402007@ntu.edu.tw (K.-Y.C.); wil1019@ntu.edu.tw (Y.-L.W.); jungtsuchen@ntu.edu.tw (J.-T.C.); ypwang0530@ntu.edu.tw (Y.-P.W.); 2 Department of Pediatric Dentistry, National Taiwan University Children’s Hospital, Taipei City 100, Taiwan; 3 Graduate Institute of Biomedical Electronics and Bioinformatics, National Taiwan University, Taipei City 106, Taiwan; d05945012@ntu.edu.tw; 4 Department of Biologic and Materials Sciences, University of Michigan School of Dentistry, Ann Arbor, MI 48108, USA; jsimmer@umich.edu (J.P.S.); janhu@umich.edu (J.C.-C.H.)

**Keywords:** hypodontia, oligodontia, tooth development, WNT signaling, genetic mutation, exome sequencing, digenic inheritance, incomplete penetrance, variable expressivity, precision medicine

## Abstract

Familial tooth agenesis (FTA), distinguished by developmental failure of selected teeth, is one of the most prevalent craniofacial anomalies in humans. Mutations in genes involved in WNT/β-catenin signaling, including *AXIN2* *WNT10A*, *WNT10B*, *LRP6*, and *KREMEN1,* are known to cause FTA. However, mutational interactions among these genes have not been fully explored. In this study, we characterized four FTA kindreds with *LRP6* pathogenic mutations: p.(Gln1252*), p.(Met168Arg), p.(Ala754Pro), and p.(Asn1075Ser). The three missense mutations were predicted to cause structural destabilization of the LRP6 protein. Two probands carrying both an *LRP6* mutant allele and a *WNT10A* variant exhibited more severe phenotypes, suggesting mutational synergism or digenic inheritance. Biallelic *LRP6* mutations in a patient with many missing teeth further supported the dose-dependence of *LRP6*-associated FTA. Analysis of 21 FTA cases with 15 different *LRP6* loss-of-function mutations revealed high heterogeneity of disease severity and a distinctive pattern of missing teeth, with maxillary canines being frequently affected. We hypothesized that various combinations of sequence variants in WNT-related genes can modulate WNT signaling activities during tooth development and cause a wide spectrum of tooth agenesis severity, which highlights the importance of exome/genome analysis for the genetic diagnosis of FTA in this era of precision medicine.

## 1. Introduction

Tooth formation is a developmental process hinged on epithelial–mesenchymal interactions that require intricate regulation of many transcription factors, morphogenic molecules, and signaling pathways [1]. Early disruptions or disturbances during this process can cause complete failure of tooth development [2]. In humans, familial tooth agenesis (FTA) is manifested by congenitally missing teeth and is one of the most prevalent developmental anomalies [3]. It can occur as isolated (non-syndromic) or in genetic syndromes such as ectodermal dysplasia. The disease is most often inherited in a dominant manner, while recessive conditions have also been documented. Clinically, FTA can show a reduced disease penetrance and a great variety of phenotypic severity even within the same family [2,3]. Hypodontia, oligodontia, and anodontia are descriptive terms used to describe levels of increasing severity of tooth agenesis: hypodontia for missing 1–5 teeth, oligodontia for 6 or more missing teeth, and anodontia for complete absence of teeth. Defects in genes involved in early tooth development have been demonstrated to cause non-syndromic FTA [4], including *MSX1* (OMIM*142983) [5], *PAX9* (OMIM*167416) [6], *AXIN2* (OMIM*604025) [7], *EDA* (OMIM*300451) [8], *WNT10A* (OMIM*606268) [9], and *LRP6* (OMIM*603507) [10,11]. As these genes also function in organogenesis other than tooth formation, their mutations can cause non-dental phenotypes in affected individuals, such as hair, nail, or sweating problems, except for *PAX9* [4]. Furthermore, it has been previously shown that mutations in these candidate genes appear to cause FTAs with distinct patterns of tooth agenesis [12,13]. However, unlike the other causative genes that have been well documented and studied, *LRP6* was more recently discovered to be associated with FTA. Due to limited cases and data availability, *LRP6*-associated FTA requires further investigation, as its associated patterns of tooth agenesis have yet to be elucidated.

*LRP6* (Low Density Lipoprotein Receptor-related Protein 6) is a gene that encodes a cell surface receptor belonging to the low density lipoprotein receptor (LDLR) family [14]. It serves as a co-receptor for WNT/β-catenin signaling, along with its *Drosophila* homolog Arrow and its closely related paralog LRP5 [15]. Biochemistry and structural biology studies have demonstrated that LRP6 can bind to various WNT ligands and secreted inhibitors of WNTs, DKKs and SOST (sclerostin) to modulate WNT/β-catenin signaling [16,17]. As WNT signaling is involved in many processes of organogenesis and tissue homeostasis, mutations in *LRP6* have been linked to various diseases [18] including coronary artery disease (CAD) [19], high bone mass (HBM) phenotypes [20], and neural tube defects (NTDs) [21]. The discovery that heterozygous *LRP6* loss-of-function mutations can cause FTA not only expands the phenotypic spectrum of *LRP6*-related disorders [10,11] but underscores the critical role of WNT signaling during tooth development in humans [1,2]. Patients carrying FTA-causing *LRP6* mutations primarily exhibit isolated tooth agenesis, although involvement of non-dental tissues has been reported in some cases, such as hair problems and facial clefts. Previously, *AXIN2* and *WNT10A*, two other players in WNT signaling, have been determined to be associated with FTA [7,9]. *AXIN2* (Axis Inhibitor 2) encodes an intracellular scaffold protein of β-catenin destruction complex and functions as a negative regulator of WNT signaling. Mutations in *AXIN2* cause oligodontia–colorectal cancer syndrome (OMIM#608615), in which affected individuals have severe oligodontia and a predisposition to colon polyps and/or colon cancer [7]. On the other hand, biallelic *WNT10A* mutations were first identified in patients with syndromes of ectodermal dysplasia, Schopf–Schulz–Passarge syndrome (SSPS, OMIM#224750), and odontoonychodermal dysplasia (OODD, OMIM#257980) [9]. However, it was later demonstrated that defects in *WNT10A* are frequently associated with tooth agenesis without or with only minor signs of ectodermal dysplasia, and that these account for a significant majority of FTA cases [22]. More recently, recessive mutations of *KREMEN1* (Kringle Domain-containing Transmembrane Protein 1, OMIM*609898), which encodes another receptor for DKK, were shown to cause a specific form of ectodermal dysplasia (ECTD13, OMIM#617392) that includes oligodontia [23]. Considering the involvement of these genes in a mutual signaling pathway during tooth development, interactions among their sequence variants might produce a spectrum of WNT signaling disturbances and underlie the highly variable expressivity frequently observed in FTA [24]. However, potential mutational interactions in FTA have not been fully explored.

In this study, we characterized four FTA families and identified an unreported *LRP6* pathogenic mutation for each of them. Two probands with severe oligodontia carried not only *LRP6* mutations but also *WNT10A* pathogenic variants, suggesting a potential mutational synergism or digenic inheritance for WNT signaling-related genes in FTA. One oligodontic patient who was compound heterozygous for two *LRP6* missense mutations also provided supporting evidence for a dose-dependent effect in *LRP6*-associated FTA. Through a literature review, we further delineated a specific pattern of tooth agenesis caused by *LRP6* loss-of-function mutations.

## 2. Materials and Methods

### 2.1. Enrollment of FTA Families

The study protocol and consent forms for human subject research were reviewed and approved by the Institutional Review Board Committee at the National Taiwan University Hospital (201605017RINC, date of approval: 13 July 2016). At the time of recruitment, all participants signed written consents following comprehensive explanation and discussion of the study content. Extra-oral, intra-oral, and radiographic examinations were conducted for phenotyping. Detailed personal and family history was obtained for pedigree construction. Non-stimulated saliva (2 mL) from each subject was collected using a Saliva DNA Collection and Preservation kit (Norgen Biotek Corp, Thorold, ON, Canada) to obtain genomic DNA for mutational analyses. All of the recruitment procedures were specified in our human study protocols and followed the Declaration of Helsinki.

### 2.2. Mutational Analyses

Genomic DNA was extracted from each saliva sample with prepIT•L2P solution (Norgen Biotek Corp) following the manufacturer’s instructions. To identify FTA-causing mutations, whole exome sequencing and analysis were performed for each proband. The SureSelect Human All Exon V6 Kit (Agilent Technologies, Cedar Creek, TX, USA) was used for library preparation, followed by 150-bp paired-end sequencing with the Illumina Novaseq 6000 system. Sequencing reads were aligned using BWA to GRCh37 (hg19) human reference sequence. Variants were called and annotated by GATK HaplotypeCaller and Ensembl VEP. Potential disease-causing mutations were identified through screening of an in-house panel of 966 genes associated with craniofacial development and anomalies (Table S1). Sanger sequencing was further performed for validation of the identified variants and analysis of their segregation within each family, using corresponding primer sets for *LRP6* and *WNT10A*. For numbering gDNA and cDNA mutation positions, the subject’s sequence variants were compared to human reference sequences NG_016168.2 and NM_002336.3 for *LRP6* and NG_012179.1 and NM_025216.3 for *WNT10A*.

### 2.3. Prediction of Structural Alterations Caused by LRP6 Mutations

To investigate the potential impact of the identified *LRP6* missense mutations on protein structure we conducted computational predictions using PremPS, recently developed software that has been shown to outperform currently available methods [25]. For prediction of p.Ser127Thr and p.Met168Arg the PDB (Protein Data Bank) structure of 3S94 was used, which constituted a crystal structure of the human LRP6 extracellular domain (E1E2). On the other hand, 4A0P, the crystal structure of LRP6-E3E4, was employed for prediction of p.Ala754Pro, p.Ser817Cys, and p.Asn1075Ser. “A chain” was selected in all predictions, and mutation specified manually. In the outputs, a predicted unfolding free energy change (ΔΔG) was calculated for each mutation, with positive and negative values respectively indicating destabilizing and stabilizing mutations. The location of the mutation (surface or core) was also provided, along with the predicted 3D structure of the mutant peptide produced by FoldX.

### 2.4. Literature Review and Statistical Analyses

Thirteen articles in English reporting on tooth agenesis phenotypes with *LRP6* mutations were identified through a systematic search of PubMed/MEDLINE and Google. All articles were scrutinized to retrieve available phenotypic data on tooth agenesis for all individuals carrying *LRP6* sequence variants. One manuscript was excluded, as it reported an interstitial deletion of 290 kb in 12p13.2, which included *LRP6* and two adjacent genes [26]. For the remaining twelve articles, data about the *LRP6* mutations and dental phenotypes of each documented individual were extracted. Correct annotation and description of the mutations was confirmed following HGVS nomenclature recommendations. For statistical analyses, we included only cases with loss-of-function *LRP6* mutations, including nonsense, frameshift, and splice-site variants. The number of missing teeth in total and in each tooth type (combining left and right, but excluding third molars) were calculated. Some cases of missense mutations were not included due to limited available phenotypic data and undetermined pathogenicity of *LRP6* variants in some reports.

## 3. Results

### 3.1. Family 1

**Figure 1:**
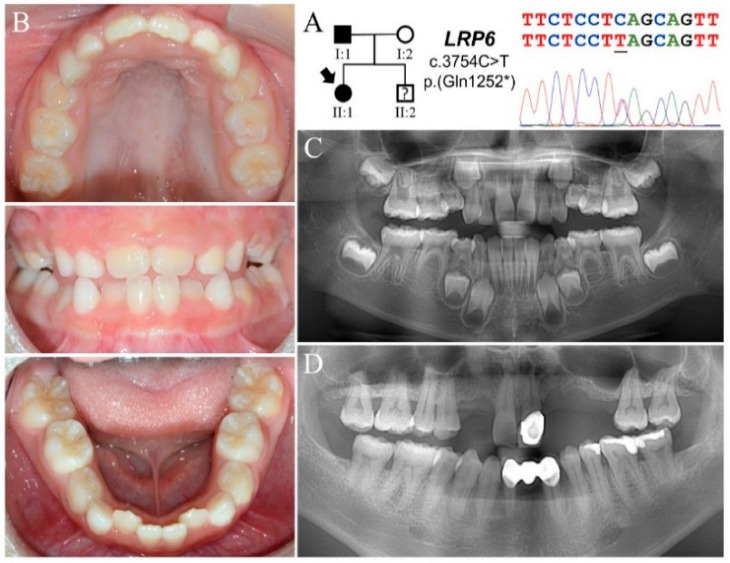
Family 1 with a novel *LRP6* p.(Gln1252*) mutation. (**A**) The Family 1 proband (II:1) inherited FTA from her father (I:1), suggesting a dominant pattern of inheritance. The DNA sequencing chromatogram shows a C-to-T transition that converts a CAG (glutamine) codon into a TAG (stop) codon in one *LRP6* allele. (**B**) The proband, age seven, presented with a mixed dentition with no apparent microdontia or tooth dysmorphology. All primary first molars appeared to be in infraocclusion. (**C**) The panoramic radiograph of the proband revealed a total of eight missing tooth germs excluding third molars. Taurodontism of primary and permanent molars was not evident. (**D**) The father had multiple missing teeth and wore a maxillary partial denture. While tooth numbers 6, 11, and 12 were previously extracted, tooth numbers 7, 10, 13, 23, 24, and 25 as well as all third molars were congenitally absent.

The proband of Family 1 (II:1) was a seven-year-old girl who inherited tooth agenesis from her father (I:1) (Figure 1A). She was otherwise healthy without heat intolerance, nail dysplasia, or hair problems. Clinically, she had a mixed dentition with mild crowding of lower anterior teeth. Both primary and permanent teeth were of normal morphology and size. All primary molars appeared infraoccluded except for tooth letters K and T (Figure 1B and Figure S1B). The panoramic radiograph revealed a total of eight missing permanent teeth (tooth numbers 4, 5, 7, 10, 12, 13, 20, 29) excluding third molars (Figure 1C and Figure S1C) (Table 1). At the time of recruitment, her father (I:1) had nine teeth missing and wore a maxillary partial denture. While tooth numbers 6, 11, and 12 had been extracted, the other six teeth were congenitally absent (Figure 1D). Her mother (I:2) had a complete permanent dentition, except for maxillary third molars and an extracted tooth (#30) replaced with a dental implant (Figure S1D). No family history of intestinal polyps or colorectal cancer was reported.

Exome analysis of the proband’s DNA identified a heterozygous C to T transition at Exon 18 of *LRP6* (NG_016168.2:g.139841C>T; NM_002336.3:c.3754C>T) (Figure 1A). This sequence variant changes a glutamine codon (CAG) to a translation termination codon (TAG) at position 1252 (NP_002327.2:p.Gln1252*) and will likely subject the altered transcript to nonsense mediated decay. The mutation is not documented in the Genome Aggregation Database (gnomAD) or the Taiwan BioBank database [27]. In addition, a missense sequence variant in *WNT10A* (NG_012179.1:g.6853G>A; NM_025216.3:c.338G>A; NP_079492.2:p.Arg113His) was also identified (Figure S1A). This variant, designated as rs749324327, has a minor allele frequency (MAF) of ~0.0004 in East Asian (EAS) populations and is predicted to be “benign”, with a PolyPhen-2 score of 0.015. No potential pathogenic mutations were detected in other candidate genes of FTA. Further Sanger sequencing and segregation analysis indicated that the *LRP6* and *WNT10A* mutations were both inherited from the father.

### 3.2. Family 2

**Figure 2:**
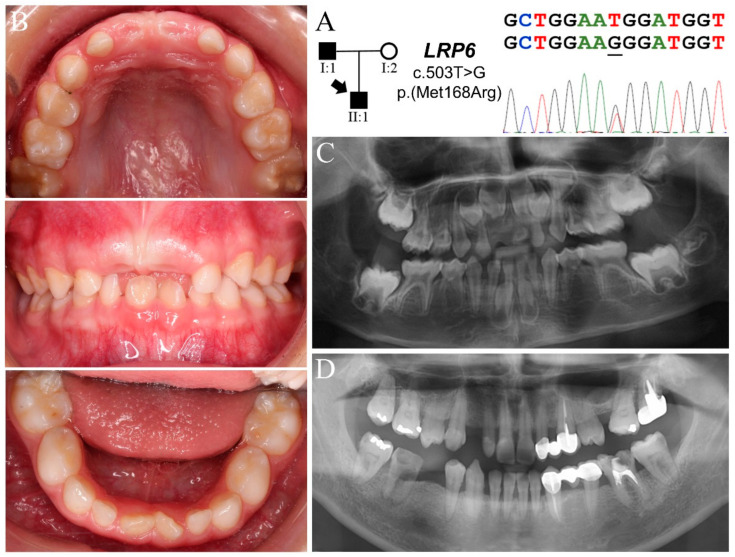
Family 2 with a novel *LRP6* p.(Met168Arg) mutation. (**A**) The pedigree indicates a nuclear family in which the proband and his father had FTA. The DNA sequencing chromatogram shows that both of them were heterozygous for an *LRP6* missense defect: g.68531T>G, c.503T>G, p.Met168Arg. (**B**) Dental phenotype of proband (age six) showing a mixed dentition with normal tooth size and shape. The enamel of tooth numbers 3 and 14 appeared hypomineralized. (**C**) Radiographically the tooth germs of thirteen permanent teeth were not detected in the proband at age five. The upper anterior tooth germs appeared to show lobodontia, exhibiting fang-like cuspids. (**D**) The father’s panorex shows that he had four missing bicuspids. The maxillary lateral incisors looked microdontic, although tooth number 10 has been restored. Tooth number 27 appeared lobodontic.

Family 2 was a nuclear family in which the proband (II:1, age six) had thirteen missing teeth (tooth numbers 4, 5, 6, 12, 13, 20, 21, 22, 23, 26, 27, 28, 29) excluding third molars (Figure 2) (Table 1). While his primary teeth were not evidently microdontic or dysmorphic, radiographically the tooth germs of maxillary permanent incisors and canines appeared slender and lobodontic (Figure 2B,C and Figure S2C). No signs of ectodermal dysplasia were clinically noted except for mild perioral dry skin and hyperpigmentation. While family history was stated to be noncontributory, the father (II:1) was found to be missing four bicuspids (tooth numbers 5, 13, 21, 29) and all third molars (Figure 2D). His teeth were generally small, particularly the peg-shaped maxillary lateral incisors. The mother (II:2) presented with all permanent teeth except maxillary third molars and extracted tooth number 15 (Figure S2D).

Analysis of the proband’s exome revealed four potential disease-causing mutations in FTA candidate genes: three heterozygous missense variants in *LRP6* (g.68531T>G, c.503T>G, p.Met168Arg; g.112084C>G, c.2450C>G, p.Ser817Cys; g.146466A>G, c.4333A>G, p.Met1445Val) and one in *WNT10A* (g.14712G>A, c.637G>A, p.Gly213Ser) (Figure 2A and Figure S2A,B). Among these four mutations, while the c.503T>G variant in *LRP6* is not listed in the databases, the other three are rare sequence variants with respective MAFs of 0.0114 (*LRP6* c.2450C>G, rs2302686), 0.0007 (*LRP6* c.4333A>G, rs761703397), and 0.0284 (*WNT10A* c.637G>A, rs147680216) in EAS. The novel *LRP6* c.503T>G mutation substitutes the hydrophobic methionine^168^ for an arginine (p.Met168Arg) and is predicted to be “probably damaging”, with a PolyPhen-2 score of 1. The other two *LRP6* variants, c.2450C>G (p.Ser817Cys) and c.4333A>G (p.Met1445Val), were considered to be “possibly damaging” and “benign”, having PolyPhen-2 scores of 0.723 and 0, respectively. On the other hand, the *WNT10A* mutation (p.Gly213Ser) is well documented to cause tooth agenesis with incomplete penetrance [28,29]. Segregation analysis of the parent-child trio revealed that the father, who was hypodontic, carried all three *LRP6* variants but not the *WNT10A* mutation, which was found in the mother. This segregation pattern of mutations suggested a plausible synergetic effect from the *LRP6* and *WNT10A* mutations, which caused thirteen missing teeth in the proband.

### 3.3. Family 3

**Figure 3:**
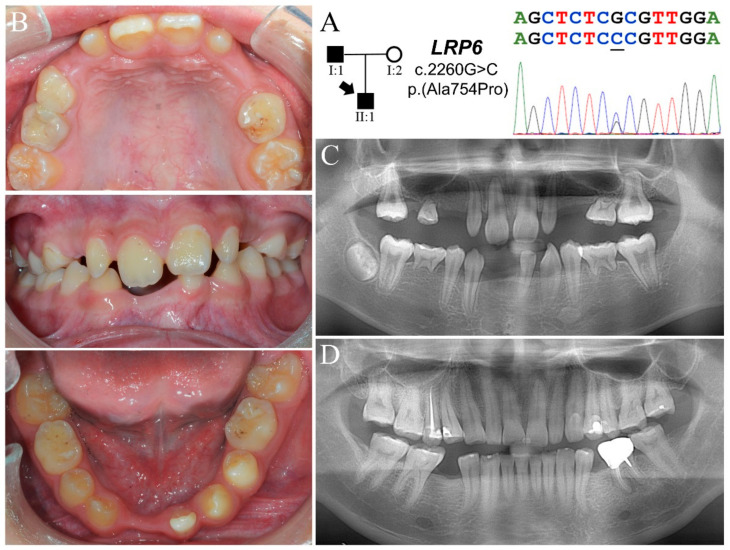
Family 3 with a novel *LRP6* p.(Ala754Pro) mutation. (**A**) The pedigree demonstrates a dominant pattern of FTA inheritance. The DNA sequencing chromatogram shows a G-C transversion that changes a GCG alanine codon into a CCG proline codon in one *LRP6* allele. (**B**) Dental phenotype of the proband (II:1, age eleven) shows a mixed dentition with five over-retained primary molars. While the primary teeth were not overtly microdontic, the permanent teeth appeared small and dysmorphic, particularly the maxillary lateral incisors. (**C**) Proband’s panoramic radiograph at age twelve shows a total of fourteen missing permanent teeth excluding third molars. All anterior teeth looked slender, and the roots of permanent molars were aberrantly convergent. (**D**) The father’s panorex shows that both of his mandibular second bicuspids were missing. Similar to the proband, he also had peg-shaped upper laterals. All of his teeth exhibited moderate to severe dental attrition.

The proband of Family 3 (II:1) was an eleven-year-old boy who had severe oligodontia (Figure 3A). According to the mother, his hair has been growing slowly since childhood, but no sweating problem was noted. Clinically, he had only thirteen permanent teeth and five over-retained primary molars (Figure 3B and Figure S3B) (Table 1). While the primary teeth looked normal in size and morphology, the permanent teeth appeared microdontic and misshapen, especially the maxillary incisors. The maxillary first molars exhibited a heart-shaped morphology, and the mandibular ones had a reduced number of cusps. Radiographically, he had a total of fourteen missing teeth excluding third molars (tooth numbers 2, 4, 5, 6, 11, 12, 13, 15, 18, 20, 24, 25, 26, 29) (Figure 3C). The development of tooth number 31 was apparently delayed. The roots of permanent first molars appeared convergent, although taurodontism was not evident. His father (I:1) was missing both mandibular second bicuspids, while his mother (I:2) had all permanent teeth excepting the four third molars and two mandibular first molars, which were extracted due to unrestorable caries (Figure 3D and Figure S3C).

Whole exome analysis for the proband detected no potential pathogenic sequence variants in known FTA-associated genes but two missense variants in *LRP6* (g.27546T>A, c.379T>A, p.Ser127Thr; g.109666G>C, c.2260G>C, p.Ala754Pro) (Figure 3A and Figure S3A). The c.2260G>C variant, not listed in any database scrutinized, is a novel mutation that will cause a p.Ala754Pro substitution, which is predicted to be “probably damaging” (PolyPhen-2 score = 1). However, the other variant, c.379T>A, is found in 569 out of 19948 EAS chromosomes (MAF = 0.0285) and is designated rs17848270. PolyPhen-2 prediction gave the resulting p.Ser127Thr substitution a score of 0 and categorized it as a benign variant. As the father carried the p.Ala754Pro variant and the mother the p.Ser127Thr, the proband was a compound heterozygote of the two *LRP6* mutations.

### 3.4. Family 4

**Figure 4:**
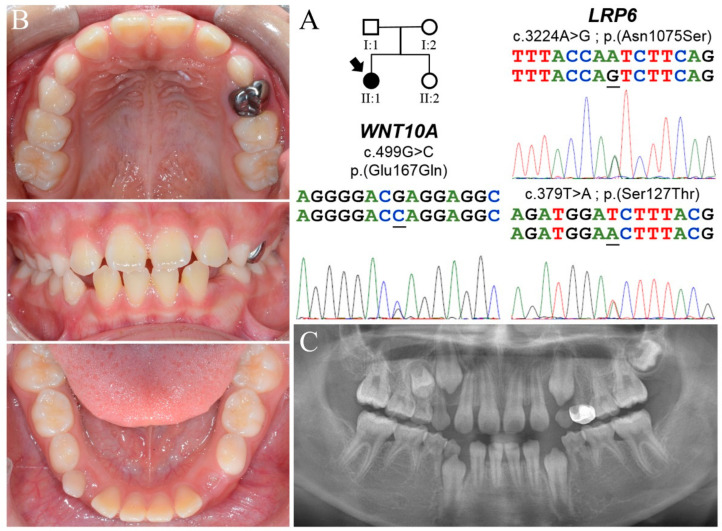
Family 4 with *LRP6* and *WNT10A* mutations. (**A**) The pedigree indicates that the proband (II:1) was a simplex case of FTA. She was the only affected individual in the family. The DNA sequencing chromatograms from the proband show two *LRP6* and one *WNT10A* heterozygous mutations. While both *LRP6* variants, p.(Ser127Thr) and p.(Asn1075Ser), were inherited from her father, the *WNT10A* mutation, p.(Glu167Gln) was maternally derived. (**B**) Dental phenotype of the proband (age ten) shows a mixed dentition with dental spacing over anterior sextants. Both primary and permanent teeth were of normal size and morphology. (**C**) Proband’s panoramic radiograph shows a total of ten missing permanent teeth, involving primarily bicuspids and second molars. The roots of the molars were not particularly convergent or taurodontic.

The proband of Family 4 (II:1) was a ten-year-old girl, who was the only individual with tooth agenesis in the family (Figure 4A). She had a mixed dentition with normal tooth size and morphology, although her permanent incisors were widely spaced (Figure 4B). She was otherwise healthy and exhibited no characteristics of ectodermal dysplasia. Her panoramic radiograph revealed ten missing permanent teeth excluding third molars (tooth numbers 2, 4, 12, 13, 18, 20, 21, 28, 29, 31) (Figure 4C) (Table 1). Maxillary first permanent molars showed mild taurodontism. While both parents had no missing teeth, their maxillary second molars appeared heart-shaped (Figure S4A,B). The father’s maxillary lateral incisors were microdontic. The younger sister (II:2) was also not affected (Figure S4C,D).

Exome analysis for the proband identified three sequence variants in FTA candidate genes, two in *LRP6* (g.27546T>A, c.379T>A, p.Ser127Thr; g.124339A>G, c.3224A>G, p.Asn1075Ser) and one in *WNT10A* (g.14574G>C, c.499G>C, p.Glu167Gln) (Figure 4A). The *LRP6* c.3224A>G mutation is a rare variant with an MAF of 0.0024 in EAS. It was predicted to be “possibly damaging”, with a PolyPhen-2 score of 0.767. The *WNT10A* mutation (c.499G>C, rs148714379), while being rare (MAF = 0.0003), was categorized as a benign variant (PolyPhen-2 score = 0.087). Segregation analysis showed that the father carried the two *LRP6* variants, while the mother and the younger sister were both heterozygotes for the *WNT10A* mutation. These results suggest that the proband’s oligodontia likely resulted from these synergistic mutations in *LRP6* and *WNT10A*.

### 3.5. Predicted Structural Alterations and Pathogenicity of LRP6 Missense Mutations

Computational prediction of the structural impact for the five *LRP6* missense mutations on protein stability demonstrated that p.Met168Arg, p.Ala754Pro, and p.Asn1075Ser were destabilizing mutations with ΔΔG values of 2.19, 1.39, and 0.96, respectively. Particularly, p.Met168Arg and p.Ala754Pro were highly destabilizing, as their ΔΔGs were higher than 1.00 kcal mol^−1^. In contrast, the other two missense variants, p.Ser817Cys and p.Ser127Thr, were predicted to stabilize the protein, having ΔΔG values of −0.6 and −0.3. Amino acid sequence alignment and analysis for ortholog proteins of LRP6 and LRP5 were also performed to evaluate the phylogenetic conservation of specific amino acids at the five mutated positions.

**Figure 5:**
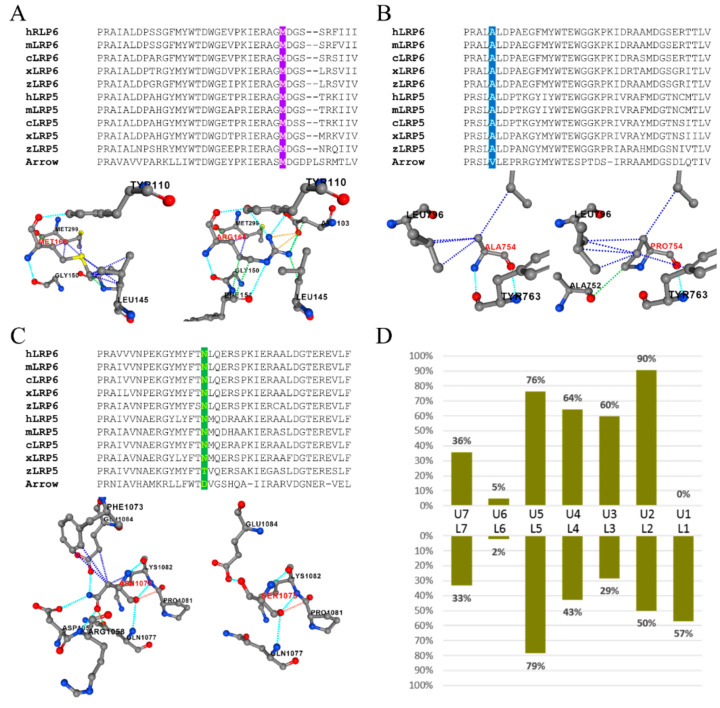
Amino acid sequence alignment and structural prediction of *LRP6* missense mutations. (**A**) Alignment of P1B3 domain (a.a. 140-177 of human LRP6). Methionine^168^ is extremely conserved throughout evolution. The p.(Met168Arg) mutation was predicted to be highly destabilizing. (**B**) Alignment of P3B3 domain (a.a. 750-787 of human LRP6). The Alanine^754^ is extremely conserved among orthologs of LRP6 and LRP5. The p.(Ala754Pro) mutation was predicted to be highly destabilizing. (**C**) Alignment of P4B3 domain (a.a. 1059-1097 of human LRP6). While Asparagine^1075^ is highly conserved among orthologs of LRP6 and LRP5, zebrafish LRP5 and *Drosophila* Arrow use threonine and aspartate, respectively, at this position. The p.(Asn1075Ser) mutation was predicted to destabilize local conformation. (**D**) Percentage of missing teeth in each tooth type of 21 patients with loss-of-function *LRP6* mutations. The missing tooth numbers from the right and left sides were pooled together. Key: U, maxillary; L, mandibular; 1, central incisor; 2, lateral incisor; 3, canine (cuspid); 4, first premolar (bicuspid); 5, second premolar (bicuspid); 6, first molar; 7, second molar.

Methionine^168^ of human LRP6 was completely conserved in all of the LRP6 and LRP5 ortholog protein sequences we scrutinized (Figure 5A). Based on PDB structure 3S94, the Met^168^ side chain interacts with Leu^145^, Pro^147^, and Met^299^ through hydrophobic interactions. Substitution of this residue with an arginine was predicted to lose the interaction with Pro^147^ and gain associations with Asp^103^ and Phe^151^ through polar or ionic interactions, which would cause a significant conformational change and destabilize the structure. Similarly, human LRP6 Alanine^754^ was extremely conserved throughout vertebrate evolution of LRP6 and LRP5, while valine is used at this position in the *Drosophila* homolog, Arrow (Figure 5B). Structurally, the p.Ala754Pro substitution altered its original interaction with Tyr^763^ and Leu^796^ and acquired an aberrant interaction with Ala^752^, which was predicted to cause destabilization. As for Asparagine^1075^, the residue was also highly conserved (Figure 5C). Its long side chain interacted with multiple surrounding amino acids. Replaced by serine, which had a shorter side chain, the residue completely lost its interaction with Asp^1057^, Arg^1058^, and Phe^1073^. Furthermore, this substitution would predictably distort the peptide backbone and further impact the structure.

Unlike the above three destabilizing mutations, the other two mutations, p.Ser817Cys and p.Ser127Thr, were predicted to have a lesser impact on protein structure. Unexpectedly, Serine^817^ remained completely conserved throughout evolution down to *Drosophila* (Figure S5A). The p.Ser817Cys mutation, while not changing the interacting residues, resulted in stronger binding with Arg^804^ and Tyr^806^, which presumably formed a tighter conformation. On the other hand, while Ser^127^ was highly conserved among all vertebral LRP6 orthologs, threonine was used at this position for most LRP5s, likely suggesting a mild structural impact from the p.Ser127Thr substitution. PremPS predicted no significant conformational change from the substitution except for an increased interaction with Asp^125^ (Figure S5B).

### 3.6. Pattern of Missing Teeth Associated with LRP6 Loss-of-Function Mutations

To investigate the phenotypic features of *LRP6*-associated FTA, we analyzed 19 reported cases from 12 articles reporting *LRP6* loss-of-function mutations with strong clinical characterization, along with our two individuals from Family 1 in this study. Overall, 15 different mutations (five nonsense, six frameshift, and four splice-site variants) from 21 subjects were identified. The number (No) of missing teeth in each subject ranged from 0 to 20, excluding third molars, with a mean of 12.48 (Figure S5C). The distribution was quite evenly spread through a range of 6~20 (standard deviation = 5.38), while a case of incomplete penetrance (No = 0) was reported for the p.(Ala383Glyfs*8) mutation. Maxillary lateral incisors were the most frequently missing tooth type with a prevalence of 90%, followed by the mandibular and maxillary second bicuspids (79% and 76%, respectively) (Figure 5D). In contrast, no maxillary central incisors were absent in the reported cases, and first and second molars were also less affected. Noticeably, maxillary canines were absent twice as frequently as mandibular canines, which is not a common feature of FTA.

## 4. Discussion

Including the four mutations we identified in this study, there have been a total of 32 different *LRP6* variants reported to be associated with FTA (Table S2). These sequence defects include missense, nonsense, frameshift, and splice-site variants and distribute over multiple exons of the gene. This mutational heterogeneity highly suggests a loss-of-function disease mechanism for *LRP6*-associated FTA. Particularly, the nonsense, frameshift, and splice-site mutations are predicted to generate null alleles, as all of them introduce a premature termination codon (PTC) prior to the last exon and would presumably trigger nonsense mediated decay (NMD) of the mutant transcript, except for the p.(Cys1532Alafs*16) mutation. However, our analyses indicated that the severity of tooth agenesis caused by these heterozygous loss-of-function *LRP6* defects varied significantly, suggesting potential influence by other genetic modifiers. Alternatively, some of these mutations might actually generate transcripts escaping NMD and produce truncated LRP6 proteins, as NMD efficiency has been shown to be modulated by factors other than the location of a PTC [30]. It has also been demonstrated that an LRP6 mutant protein with only an extracellular domain or without the cytoplasmic tail acts as a dominant negative receptor for canonical WNT signaling [15]. Therefore, truncated LRP6s generated by those mutations might interfere with the wild-type receptor generated from the normal allele and cause a more severe disease phenotype through dominant negative effects, rather than simple haploinsufficiency. This hypothesis is supported by the dental phenotype of a reported case carrying the p.(Cys1532Alafs*16) mutation, which theoretically would not trigger NMD [11]. The patient had a relatively severe phenotype of oligodontia, with absence of four primary and seventeen permanent teeth. However, there seems to be no apparent genotype-phenotype correlation between the PTC location and the disease severity, as both 5′ and 3′ PTCs are associated with a wide range of missing tooth numbers. Moreover, our analysis indicated that loss-of-function *LRP6* mutations frequently caused agenesis of the maxillary lateral incisors and all second bicuspids, while first and second molars were relatively unaffected. In particular, missing maxillary canines were also frequently observed, which is an uncommon feature of FTA. Interestingly, patients with only agenesis of the maxillary permanent canines have previously been reported to carry heterozygous *WNT10A* mutations [28], suggesting a specific role for WNT signaling in maxillary canine formation. As for the *LRP6* missense mutations, while they seem to disperse over the whole extracellular domain of LRP6, they appear to locate only at specific structural regions. The LRP6 ectodomain comprises four tandem pairs of YWTD-β-propeller-EGF-like domain (P1E1 to P4E4), followed by three LDLR type A domains [16]. Each β-propeller is a six-bladed structure that serves as a platform for protein–protein interactions. Almost all FTA-associated missense mutations are located at the third, fourth, or fifth blade of each β-propeller, which have been shown to constitute the critical interacting surfaces with various ligands of LRP6 [31,32]. Furthermore, previous crystallographic and antibody studies have demonstrated that different molecules, including WNT proteins and their inhibitors, preferentially bind to distinct regions on LRP6 [16,17]. For example, while many WNTs, such as WNT1, interact with the P1 propeller, WNT3 specifically binds to the P3 domain. This complexity of preferential binding might partly explain the high heterogeneity of disease expressivity and missing tooth patterns in FTAs caused by *LRP6* missense mutations.

It has been well documented that FTA-causing mutations can show incomplete penetrance and variable expressivity [3]. Particularly, heterozygous *WNT10A* mutations have been known to cause mild hypodontia and sometimes no missing teeth [29,33]. In this study, we demonstrated that this heterogeneity in penetrance and expressivity might result from a synergistic effect from multiple mutations or digenic inheritance. The proband of Family 2, who carried three *LRP6* variants and *WNT10A* p.(Gly213Ser) mutation, exhibited a much more severe disease phenotype, including severe oligodontia, perioral dryness, and hyperpigmentation, than that of his father, who had only the *LRP6* variants and hypodontia, suggesting a potential mutational synergism on disease expressivity. Among the three *LRP6* variants, the p.(Met168Arg) and p.(Ser817Cys) mutations both substitute an extremely conserved amino acid and potentially have a significant impact on the structure of the LRP6 ectodomain, which causes the disease. However, as all three *LRP6* variants were inherited from the father, it is possible that tooth agenesis is caused by this mutant *LRP6* allele as a whole rather than by individual mutations. On the other hand, the *WNT10A* p.(Gly213Ser) mutation has been shown to cause hypodontia or no tooth agenesis in heterozygous carriers [28,33]. The mother who passed this mutation had a full set of permanent dentition except for maxillary third molars. However, when combined with the *LRP6* mutations, it led to a severe phenotype of thirteen missing teeth in the proband. This genetic synergism is also supported by the potential digenic inheritance of *LRP6* and *WNT10A* mutations in Family 4. The proband, who had *LRP6* p.(Asn1075Ser), p.(Ser127Thr), and *WNT10A* p.(Glu167Gln) variants, showed ten missing teeth, while her parents, who passed individual mutant alleles, had no missing teeth but microdontia and dysmorphology of specific teeth. The *LRP6* p.(Asn1075Ser) mutation substitutes highly-conserved asparagine with serine, which is predicted to destabilize the protein structure. However, this residue localizes at the P4E4 domain, which has been shown to have higher variability when compared with the other three PE pairs and no known interacting proteins [16,17]. Therefore, this mutation alone might not be sufficient to cause tooth agenesis. Nevertheless, when it occurs with a defect in *WNT10A*, which functions in the same pathway, a combination of deficiencies results in a severe disease phenotype. This phenomenon of “synthetic lethality” has long been described in genetics and thought to be implicated in the molecular pathogenesis of digenic inheritance in genetic disorders [34,35]. In this study, we also showed that a second *LRP6* variant in *trans* might potentially modify the expressivity of a primary “driver” mutation. The proband of Family 3 was a compound heterozygote of p.(Ala754Pro) and p.(Ser127Thr) mutations and had fourteen missing teeth, while his father, who carried the p.(Ala754Pro) mutation, exhibited only two. Interestingly, the mother was homozygous for the p.(Ser127Thr) mutation and had all permanent teeth excepting third molars, suggesting that the variant might serve as a putative genetic modifier while not being disease-causing itself. These observations of digenic inheritance and genetic modification are supported by the significant role of WNT signaling in tooth development and a direct molecular interaction between WNT10A and LRP6. As mutations in several other genes involved in WNT signaling have also been shown to cause genetic disorders featured by tooth agenesis, such as *AXIN2* [7] and *KREMEN1* [23], it is plausible to speculate that various combinations of sequence variants in these genes could generate a range of WNT signaling activity and cause a wide spectrum of severity in tooth agenesis.

Advances in sequencing technology allow us to identify sequence variant combinations that were previously difficult to find [36,37]. It also facilitates discerning the genetic etiology of disorders that are potentially multigenic and do not follow simple Mendelian inheritance [38]. FTA, while primarily inherited in a dominant manner, frequently shows remarkably variable penetrance and expressivity. Our study suggests a plausible molecular explanation of mutational synergism for these observations, and highlights the significant role of exome/genome analysis in unravelling disease-causing mutations of FTA in the era of precision medicine.

## 5. Conclusions

Mutational synergism of different WNT signaling related genes can underlie the variable disease severity in human tooth agenesis, and should be considered in the genetic diagnosis of FTA.

## Figures and Tables

**Table 1:** Summary of the missing teeth in the FTA individuals in this study.

Family	Subject		8	7	6	5	4	3	2	1	1	2	3	4	5	6	7	8	No	LRP6 Mutation	WNT10A Mutation
Family 1	I:1	Max	X					E	X			X	E	E	X			X	6	c.3754C>T p.(Gln1252*)	c.338G>A p.(Arg113His)
		Man	X							X	X	X						X			
	II:1	Max	?			X	X		X			X		X	X			?	8		
		Man	?			X									X			?			
Family 2	I:1	Max	X				X		P						X			X	4	c.503T>G p.(Met168Arg)	-
		Man	X			X								X				X			
	II:1	Max	?			X	X	X						X	X			?	13		c.637G>A p.(Gly213Ser)
		Man	?			X	X	X	X			X	X	X	X			?			
Family 3	I:1	Max	X						P			P						X	2	c.2260G>C p.(Ala754Pro)	-
		Man	X			X									X			X			
	II:1	Max	?	X		X	X	X	P			P	X	X	X		X	?	14		
		Man	?			X			X	X	X				X		X	?			
Family 4	II:1	Max	?	X		X								X	X			?	10	c.3224A>G p.(Asn1075Ser)	c.499G>C p.(Glu167Gln)
		Man	?	X		X	X							X	X		X	?			

Number of missing teeth (No) was calculated excluding third molars. Key: Max, maxillary; Man, mandibular; X: congenital missing tooth; E: extracted tooth; P: peg-shaped lateral incisor; ?: undetermined.

## Data Availability

Whole exome sequencing data and analysis as well as additional data used to support the findings of this study are available from the corresponding author upon request.

## Supplementary Materials

The following materials are available online at https://www.mdpi.com/article/10.3390/jpm11111217/s1, Figure S1: Family 1 and a *WNT10A* p.(Arg113His) mutation; Figure S2: Family 2 and mutations of *LRP6* and *WNT10A*; Figure S3: Family 3 and mutations in *LRP6*; Figure S4: Phenotypes of Family 4 members; Figure S5: Amino acid sequence alignment and structural prediction of *LRP6* missense mutations; Table S1: Panel of 966 genes associated craniofacial development and anomalies; Table S2: Known *LRP6* mutations associated with FTA. References [39,40,41,42,43,44,45,46,47] are cited in the supplementary materials.
