# Supplementary material for: Synergistic Mutations of LRP6 and WNT10A in Familial Tooth Agenesis
Source: J Pers Med. 2021 Nov 17;11(11):1217. doi: 10.3390/jpm11111217 (PMC8621929; doi:10.3390/jpm11111217)
Supplement: Supplementary file 1 [file jpm-11-01217-s001.zip › jpm-1475506-supplementary.pdf]

Journal of Personalized Medicine

Supplementary Materials

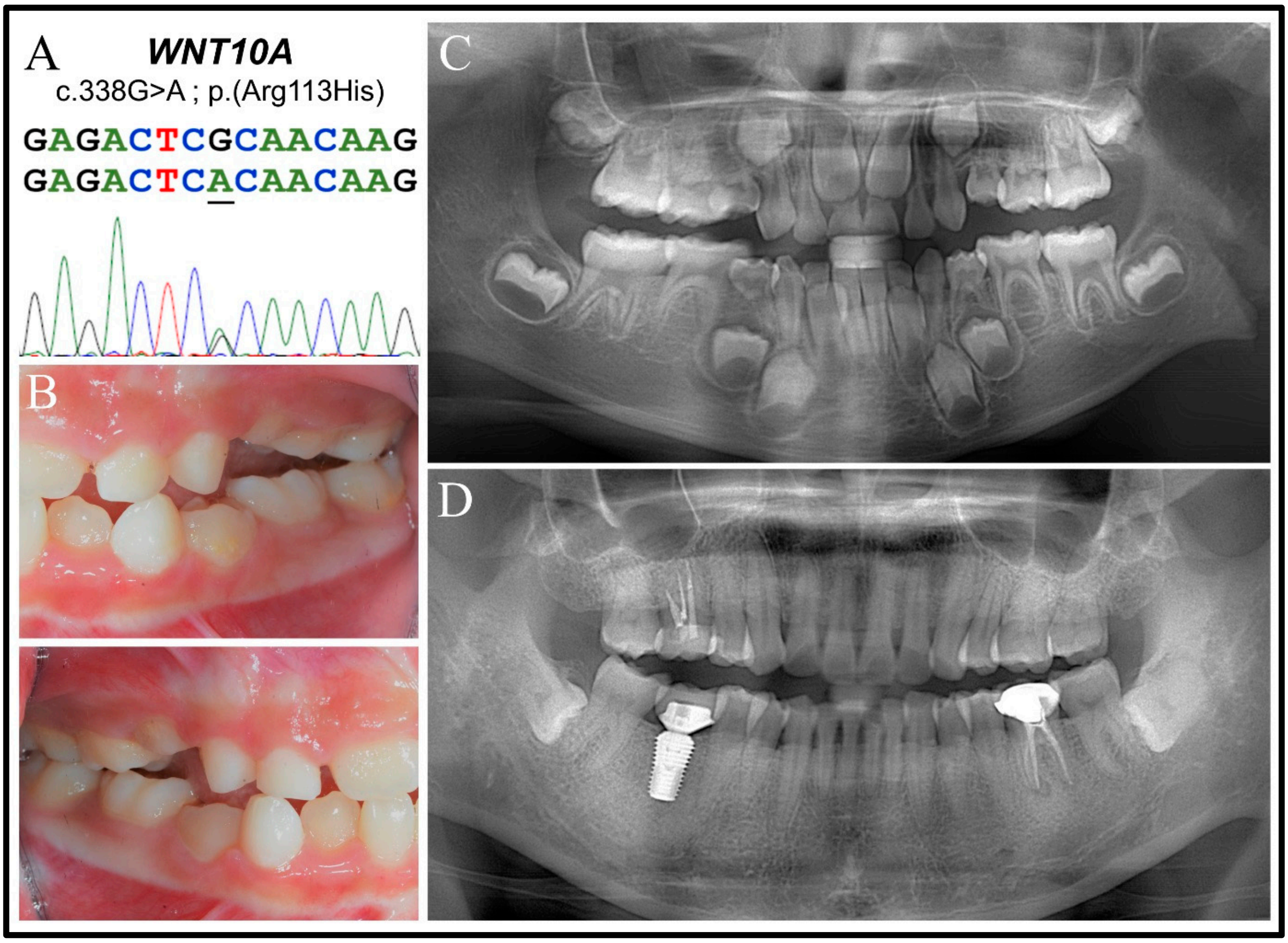

Figure S1. Family 1 and a *WNT10A* p.(Arg113His) mutation. (**A**): In addition to the *LRP6* p.(Gln1252*) mutation, the family 1 proband and her father also carried a *WNT10A* p.(Arg113His) variant (rs749324327). The DNA sequencing chromatogram shows a G-to-A transition that converts a CGC (arginine) codon into a CAC (histidine) codon in one *WNT10A* allele. (**B**): Bilateral buccal view of the proband's dentition shows that all her primary first molars were of infraocclusion. (**C**): The panoramic radiograph of the proband at age 6.5 revealed that her tooth development was not evidently delayed. (**D**): The mother's panorex shows that she had a full set of permanent teeth except for maxillary third molars and tooth number 30, which was previously extracted and restored with a dental implant.

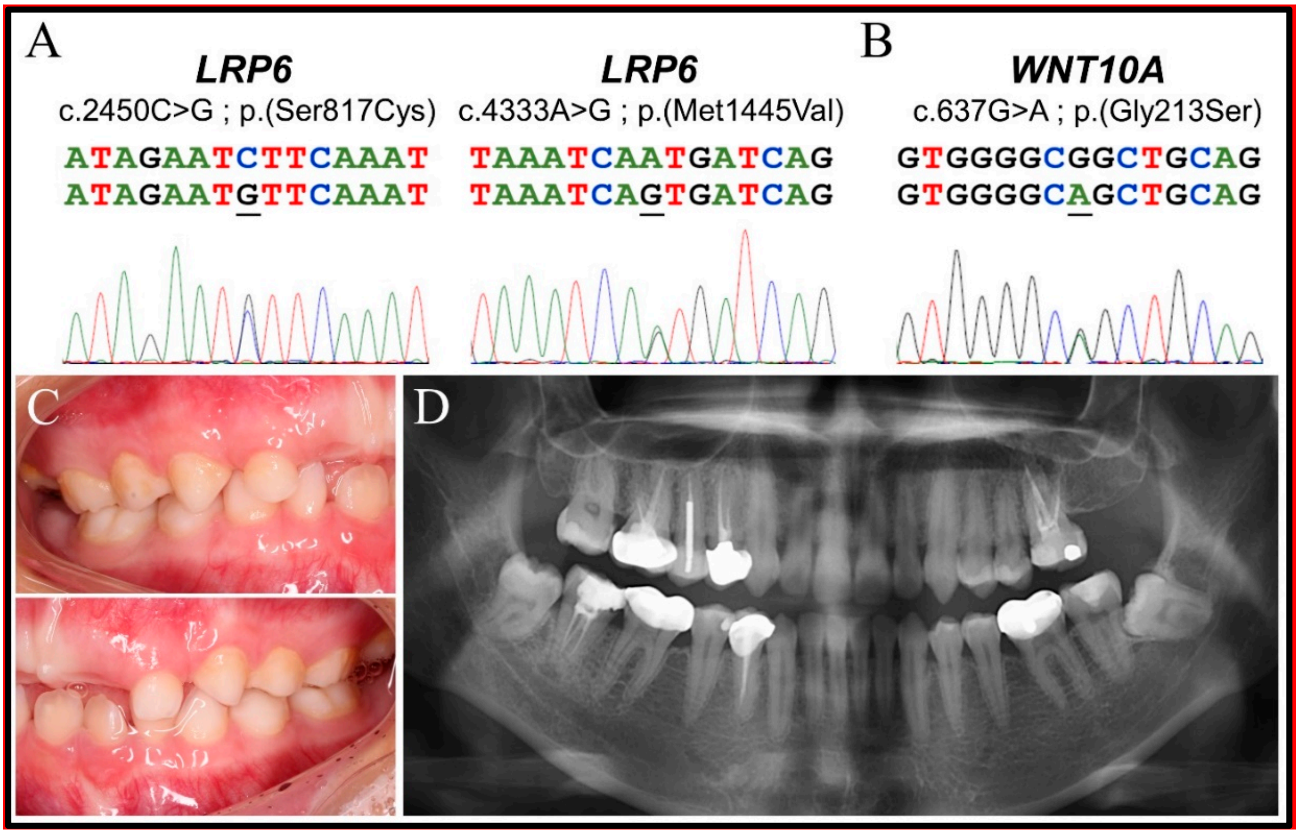

Figure S2. Family 2 and mutations of *LRP6* and *WNT10A*. (**A**)**:** In addition to the *LRP6* p.(Met168Arg) mutation, the proband and his father also carried another two *LRP6* missense variants: g.112084C > G, c.2450C > G, p.(Ser817Cys); g.146466A > G, c.4333A > G, p.(Met1445Val). This segregation pattern indicates that all these three mutations are on the same defective LRP6 allele from the paternal side. (**B**)**:** Besides *LRP6* mutations, the proband also carries a pathogenic *WNT10A* mutation inherited from his mother: g.14712G > A, c.637G > A, p.(Gly213Ser). (**C**)**:** Bilateral buccal view of the proband's dentition at age of 6. (**D**)**:** The mother's panorex shows that she had all permanent teeth except for maxillary third molars and tooth number 15, which was previously extracted due to unrestorable decay.

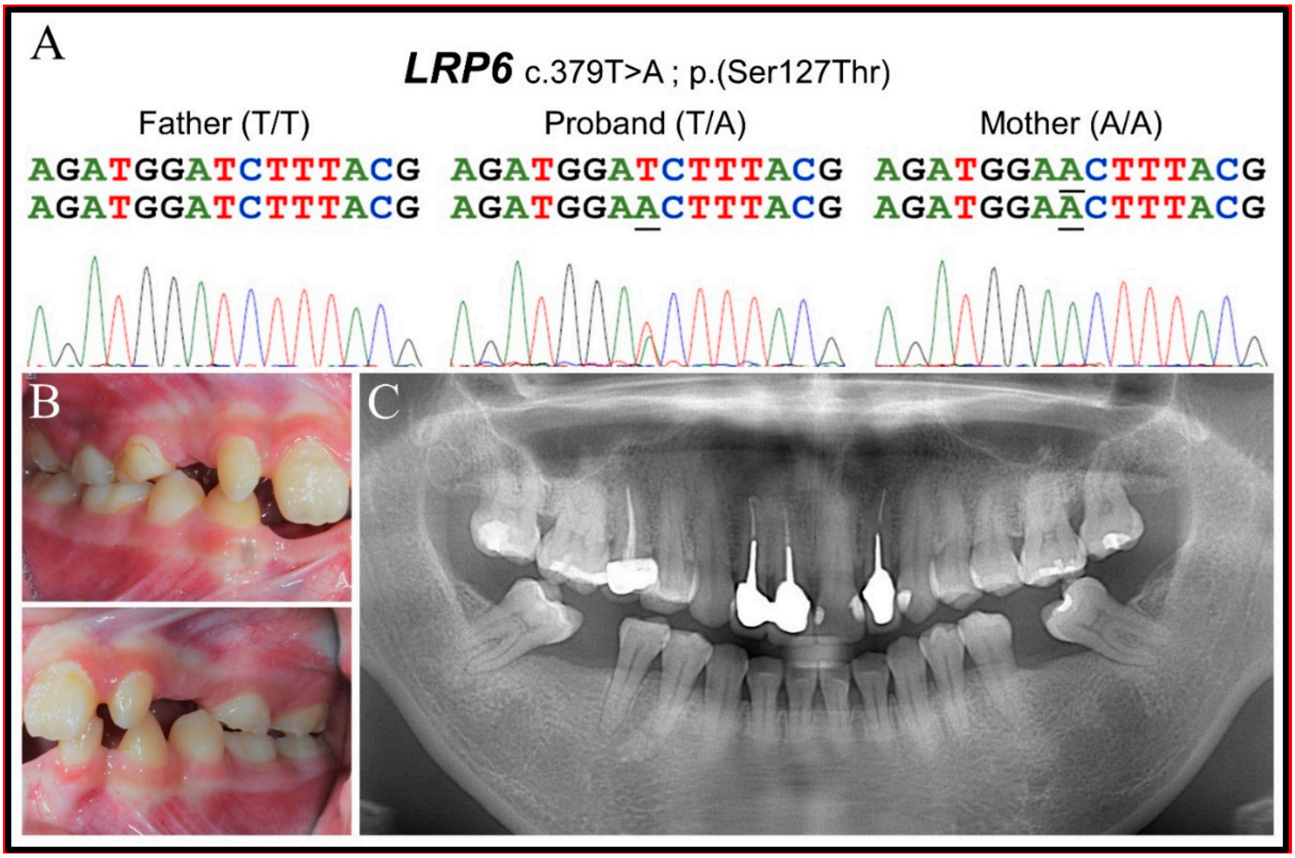

Figure S3. Family 3 and mutations in *LRP6*. (**A**)**:** In addition to the *LRP6* p.(Ala754Pro) mutation, the proband and his mother also carried another *LRP6* missense variant: g.27546T > A, c.379T > A, p.(Ser127Thr). While the father was a wild-type at this position, the proband and the mother were respectively heterozygous and homozygous to this variant. (**B**)**:** Bilateral buccal view of the proband's dentition at age of 11 shows peg laterals and slender central incisors. (**C**)**:** The mother's panorex shows that she had no missing teeth except for all the third molars and extracted mandibular first molars leading to mesial tilting of the second molars.

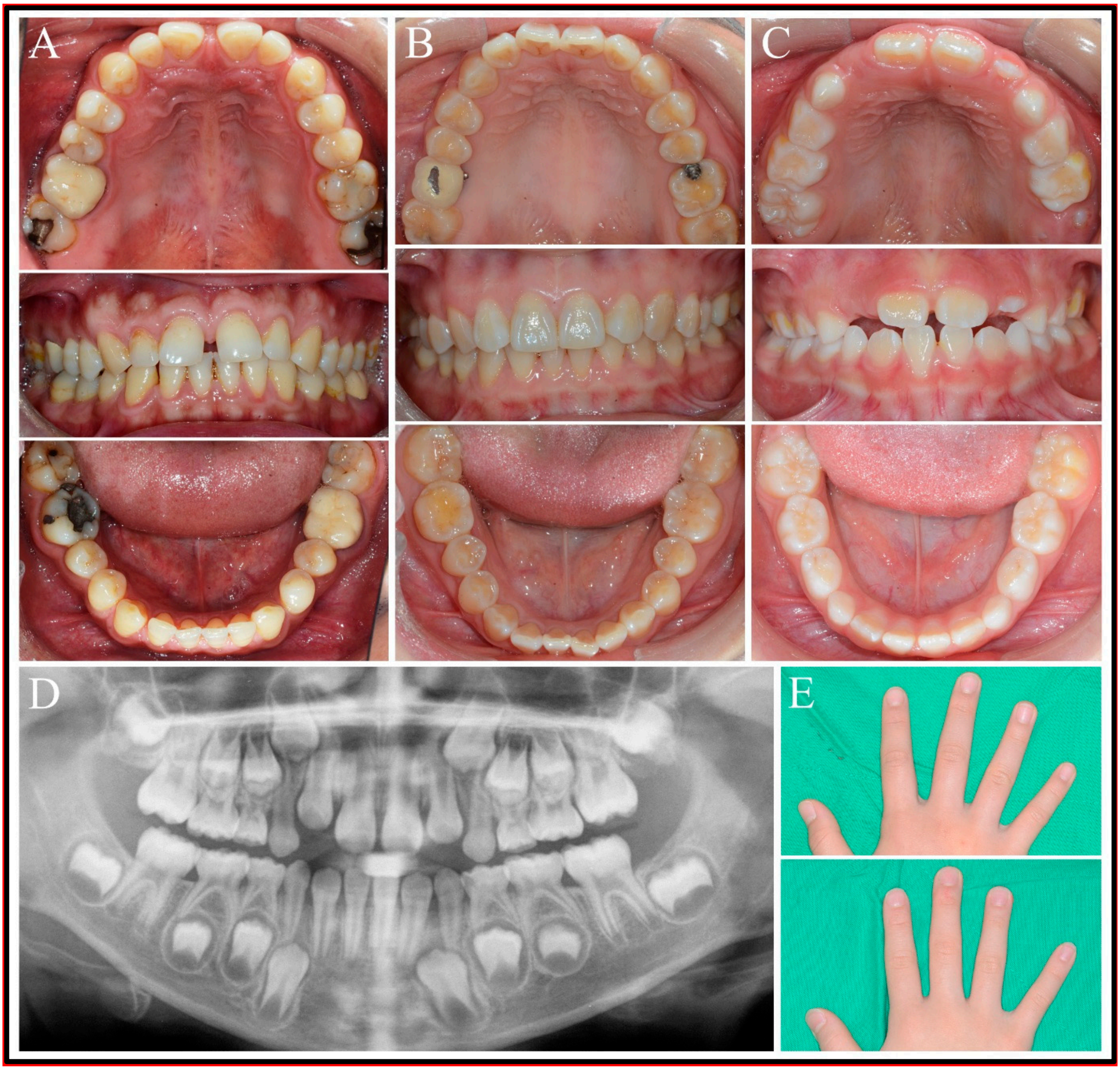

Figure S4. Phenotypes of Family 4 members. (**A**): Proband's father (age 44) had a full set of permanent dentition excluding third molars. He had maxillary central diastema with a prominent labial frenum. His upper lateral incisors were slightly microdontic, and tooth number 2 was of heart shape. (**B**): The mother at age 41 had no congenital missing teeth. Her teeth were of normal size but exhibited moderate to severe attrition. Tooth numbers 2 and 14 were both heart-shaped with only one palatal cusp. (**C**): Proband's younger sister at age 7.5 had a mixed dentition with normal tooth size and morphology. (**D**): The younger sister's panorex shows that she had all permanent tooth germs excluding third molars. (**E**): Photographs of proband's (upper panel) and her sister's (lower panel) fingers exhibit no apparent nail abnormalities.

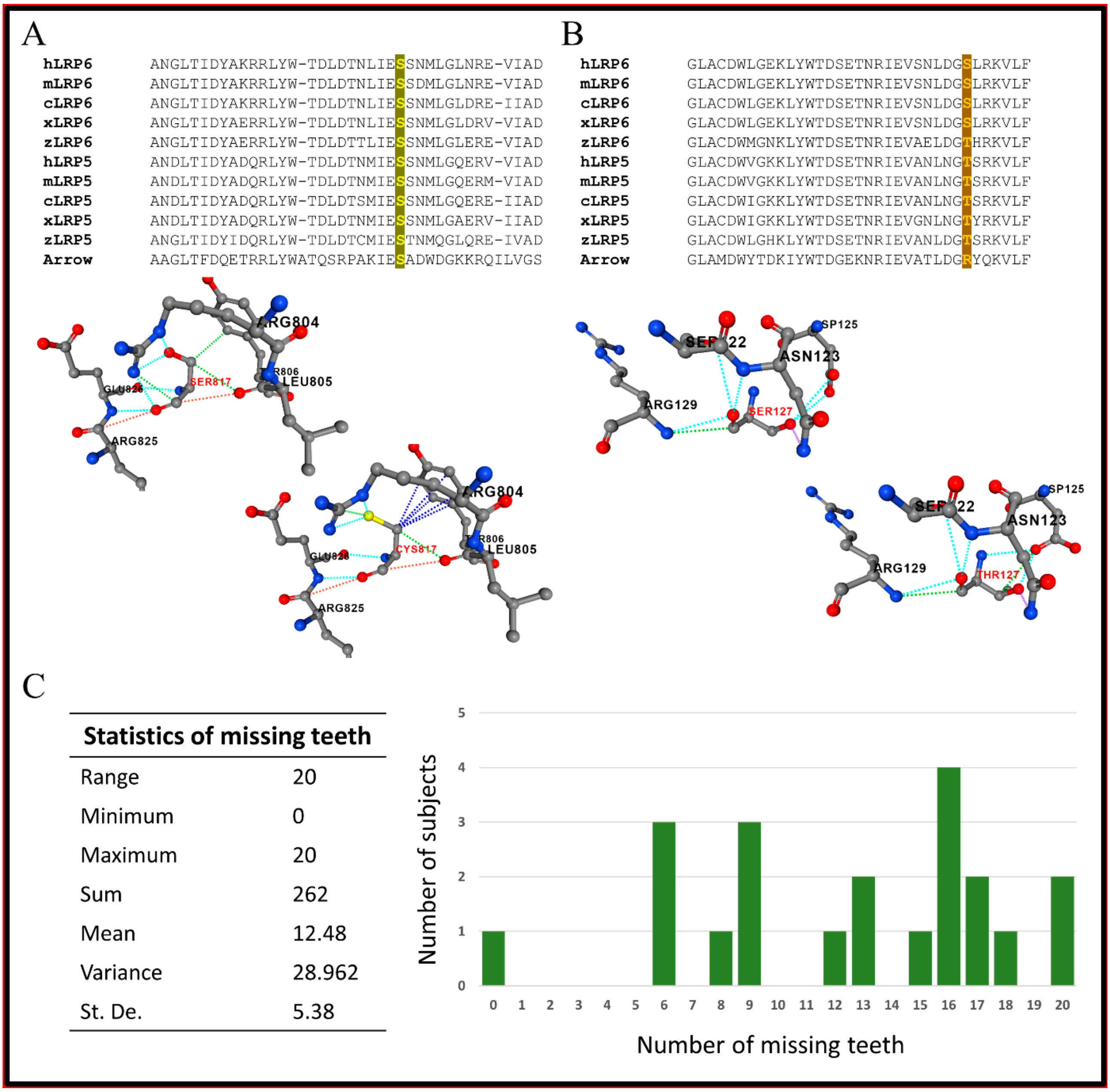

Figure S5. Amino acid sequence alignment and structural prediction of LRP6 missense mutations. (**A**): Alignment of P3B4 domain (a.a. 793–830 of human LRP6). The Serine[817] is extremely conserved throughout evolution. The p.(Ser817Cys) mutation was predicted to be stabilizing. (**B**): Alignment of P1B2 domain (a.a. 99–133 of human LRP6). The Ser[127] is conserved among orthologs of LRP6. The p.(Ser127Thr) mutation was predicted to stabilize local conformation. (**C**): Statistics of missing teeth from 21 subjects with *LRP6* loss-of-function mutations.

Table S1. Panel of 966 genes associated craniofacial development and anomalies.

AIRE ALPL ALX3 AMBN AMELX AMELY AMTN ANKRD11 ANTXR1 APC ATR AXIN2 BCOR BLM BMP4 C4orf26 CA2 CCBE1 CCDC8 CEP152 CFDP1 CHD7 CLCN7 CLDN1 CNNM4 COL17A1 COL1A1 COL1A2 COL5A1 COL5A2 COL7A1 COL9A1 COL9A2 COX7B CREBBP CRTAP CTNNB1 CTSC CTSK CUL7 CYP27B1 DLX3 DSP DSPP EDA EDAR EDARADD ELN ENAM EP300 EVC EVC2 EXT1 FAM20A FAM83H FERMT3 FGF10 FGF23 FGF3 FGF8 FGFR1 FGFR2 FGFR3 FKBP10 FOXC1 GALNT3 GAS1 GDF5 GJA1 GLA GLI2 GNAS GTF2I GTF2IRD1 IFT43 IKBKG IL11RA IRF6 KAL1 KANSL1 KDM6A KLK4 KMT2D KRT14 KRT5 LAMA3 LAMB3 LAMC2 LEF1 LEMD3 LEPRE1 LIMK1 LRP5 LTBP3 MID1 MMP1 MMP20 MSX1 MSX2 MUTYH NFKBIA NHS NSD1 OBSL1 ODAM OFD1 ORAI1 OSTM1 PAX9 PCNT PHEX PITX2 PLEC PLEKHM1 PLXNB2 POLR1C POLR1D POLR3A POLR3B PORCN PPIB PRKAR1A PROK2 PROKR2 PTCH1 PTCH2 PTH1R PVRL1 PVRL4 RAB23 RAI1 RASGRP2 RBBP8 RECQL4 RFC2 ROGDI ROR2 RUNX2 SALL4 SAT1 SATB2 SEC23A SERPINF1 SERPINH1 SH3BP2 SHH SIX3 SLC24A4 SLC26A2 SMARCAL1 SMOC2 SP6 SP7 SPARC SPARCL1 SUFU SUMO1 TBX22 TCIRG1 TCOF1 TGIF1 TNFRSF11A TNFRSF11B TP63 TRPS1 TSC1 TSC2 TUFT1 UBR1 VDR WDR72 WNT10A WNT5A WRN ZIC2
ACVR1 ACVR2A ACVR2B ADAMTS10 ADAMTS2 ADARA AGPAT2 AGXT AHCY AIP ALDH3A2 ALKBH1 AMER1 ANKH AP2B1 AP3B1 APAF1 APCDD1 ARHGAP6 ARSB ASXL1 ATP6V0A2 ATP7A ATRIP ATRX B3GALTL B3GAT3 B4GALT7 BANF1 BARX1 BCL11B BGLAP BGN BMP2 BMP7 BMPR1A BNC2 BOC BRAF CACNA1C CARD9 CASK CASP7 CCL2 CD96 CDC42 CDC6 CDH1 CDH23 CDH3 CDKN1A CDKN1C CDON CDSN CENPJ CHRD CHST3 CHSY1 CHUK CIB2 CLCN5 CLEC7A COL10A1 COL11A1 COL11A2 COL2A1 COL3A1 COX4I2 CRISPLD2 CRK CSF1 CTGF DCAF17 DCN DFNB31 DHCR24 DHCR7 DHODH DKC1 DKK1 DKK4 DLG1 DLL1 DLX1 DLX2 DLX4 DLX5 DLX6 DMP1 DPYSL4 DSC3 DSG4 EDNRB EFNB1 EHMT1 EXT2 EXTL3 EYA1 FAM20C FBN1 FERMT1 FGD1 FGF13 FGF18 FGF20 FGF4 FGF9 FKBP6 FLNA FLNB FMOD FOS FOXE1 FOXF2 FOXO1 FRAS1 FREM2 FRZB FST FUZ FZD1 FZD2 FZD6 G6PC3 GAB1 GAD1 GALNS GDNF GJB6 GLB1 GLI1 GLI3 GNPTAB GORAB GPC3 GRB2 GSC GSK3B GUSB HAND1 HAND2 HCCS HDAC4 HGSNAT HHAT HOXC13 HOXD13 HR HRAS HYAL IBSP ICAM1 IDS IDUA IFT122 IFT88 IGF1 IHH IL17F IL17RA INHBA INHBB INPP5E INSR ISL1 ITGA11 ITGA6 ITGAV ITGB2 ITGB4 ITGB6 ITGB8 JAG1 JAG2 KAT6B KAZN KCNJ2 KIF7 KISS1 KISS1R KL KRAS KRT1 KRT10 KRT16 KRT17 KRT6A KRT6B KRT74 KRT83 KRT9 LAMA5 LHX6 LHX8 LIFR LIPH LMNA LOR LPAR6 LRP4 LRP6 LTBP2 LUM LUZP1 MAP2K1 MAP2K2 MAP3K11 MASP1 MBTPS2 MED12 MED25 MEPE MGP MITF MMP14 MMP16 MMP2 MMP3 MMP9 MN1 MNT MSC MVP MYO7A NAGLU NCOA2 NCOR1 NELF NF2 NFE2L2 NFIC NIPBL NKX2-3 NKX3-2 NLRP1 NOG NOP10 NOTCH1 NOTCH2 NOTCH3 OCRL ORC1 OSR2 PAF1 PCDH15 PDGFA PDGFC PDGFRA PDS5A PDS5B PHC1 PIGL PITX1 PKDCC PKP1 PLCD1 PLG PLOD1 PLOD3 PLXNA1 PLXNA2 PLXNA3 PLXNA4 PLXNB1 PLXNB3 PLXNC1 PLXND1 POLD1 POSTN POU1F1 PRDM1 PRDM16 PRKCI PRRX1 PRRX2 PTHLH PTPN11 PTPRF PTPRS PVRL3 RAPSN RBL1 RBL2 RBM28 RFNG RGS2 RIN2 RMRP RPS6KA3 RSPO2 RSPO4 SCARF2 SFN SGSH SH3PXD2B SHOX2 SIM2 SLC20A2 SLC32A1 SLC34A2 SLC35B2 SLC35C1 SLC39A13 SLC4A2 SLC4A4 SLC4A5 SMAD2 SMAD3 SMAD5 SMG1 SMO SMPD3 SNAI1 SNAI2 SOS1 SOST SOSTDC1 SOX10 SOX11 SOX18 SOX2 SOX3 SOX5 SP3 SPP1 SPRY2 SPRY4 SQSTM1 SSTR5 ST14 STAG1 STAT1 STAT3 STIM1 SUOX TAB2 TACR3 TBCE TBX1 TBX10 TBX15 TBX2 TBX3 TCF21 TCP1 TERC TERT TFAP2A TFIP11 TGFA TGFB1 TGFB2 TGFB3 TGFBR1 TGFBR2 THRA TINF2 TMCO1 TMEM107 TNFRSF19 TNFSF11 TRAF6 TRIM37 TRIP11 TRPV3 TSHZ1 TWIST1 TWIST2 UBB USH1C USH1G USH2A WDR19 WDR35 WHSC1 WNT1 WNT10B WNT3 WNT4 WNT6 WNT7B ZEB1 ZEB2 ZMPSTE24 ZNF469
ADAM10 AKAP9 ANTXR2 BAZ1B BBX BMP1 CHPF CSRP2BP FAM111A FAM111B FAM73B GALC HMX3 IFITM5 IFT20 ITGA3 ITGB1 KDM4B LTBP1 NSUN2 NTRK1 RHOBTB3 SLC25A21 TMEM38BUBE3B
EGFR NR2F1 PAX6 PDGFRB PTHR1 ALX1 SMAD6 EGF ERBB3 FGF1 ERBB4 ERBB2 FGF5 ACVR1B MET HOXD10 CAV1 TFAP2C FGF7 BMP6 CBLB BMPR2 BMP5 EPHB3 IGF1R NTRK3 PAX3 FGF2 FGF6 RUNX3 IRAK3 BMPR1B TP53 LYN TCF7L1 CDKN1B CHX10
GPR68 ACPT ACP4 ODAPH RELT KREMEN1 KREMEN2 TSPEAR PEX1 PEX2 PEX6 SLC13A5 CLDN16 GPR98 SLC10A7 CLDN4 CLDN10 CLDN19 CTBP1 PTDSS1 NRAS CEP78 TANGO1 REST KCNQ1 KCNMA1 KCNN3 KCNH1 ATP6V1B2 KCNK4

*This gene list is modified from an NGS gene panel that targets known and candidate genes in orodental disease [1].

Table S2. Known *LRP6* mutations associated with FTA.

#	Exon	Gene (NG_016168.2)	cDNA (NM_002336.3)	Protein (NP_002327.2)	Ref
1	2	g.27223C>T	c.56C>T	p.(Ala19Val)	[2]
2	2	g.27362dup	c.195dup	p.(Tyr66Ilefs*4)	[3]
3	3	g.68531T>G	c.503T>G	p.(Met168Arg)$	Family 2
4	3	g.68545C>G	c.517C>G	p.(Arg173Gly)	[4]
5	3	g.68581A>C	c.553A>C	p.(Asn185His)	[5]
6	3	g.68630C>T	c.602C>T	p.(Ala201Val)	[5]
7	4	g.84789_84795delinsA	c.678_684delinsA	p.(His226_Phe228delinsGln)	[6]
8	4	g.84822G>T	c.711G>T	p.(Leu237Phe)	[7]
9	6	g.90465C>T	c.1003C>T	p.(Arg335*)	[8]
10	6	g.90557dup	c.1095dup	p.(Asp366Argfs*13)	[3]
11	6	g.90606_90607dup	c.1144_1145dup	p.(Ala383Glyfs*8)	[2]
12	7	g.91929C>T	c.1406C>T	p.(Pro469Leu)	[4]
13	8	g.106640A>T	c.1603A>T	p.(Ile535Leu)	[9]
14	8	g.106646G>A	c.1609G>A	p.(Gly537Arg)	[4]
15	8	g.106718C>T	c.1681C>T	p.(Arg561*)	[3]
16	9	g.107332dup	c.1779dup	p.(Glu594*)	[2]
17	9	g.107477dup	c.1924dup	p.(Ile642Asnfs*11)	[10]
18	10	g.109630_109631dup	c.2224_2225dup	p.(Leu742Phefs*7)	[2]
19	10	g.109666G>C	c.2260G>C	p.(Ala754Pro)&	Family 3
20	11	g.111926G>A	c.2292G>A	p.(Trp764*)	[3]
21	12	g.112828G>A	c.2570G>A	p.(Arg857His)	[11]
22	12	g.113005G>T	c.2747G>T	p.(Cys916Phe)	[8]
23		g.121043G>A	c.2994+1G>A	p.(?)	[4]
24	14	g.122806C>T	c.3076C>T	p.(Arg1026Cys)	[9]
25	15	g.124339A>G	c.3224A>G	p.(Asn1075Ser)#	Family 4
26	15	g.124488C>T	c.3373C>T	p.(Arg1125*)§	[12]
27	15	g.124503C>T	c.3388G>A	p.(Asp1130Asn)	[9]
28		g.133342A>C	c.3398-2A>C	p.(?)	[4]
29		g.133556_133559del	c.3607+3_3607+6del	p.(?)	[13]
30	18	g.139841C>T	c.3754C>T	p.(Gln1252*)	Family 1
31		g.144955A>G	c.4082-2A>G	p.(?)	[4]
32	23	g.150504del	c.4594del	p.(Cys1532Alafs*16)	[4]

$This mutation was identified in an *LRP6* mutant allele with additional p.(Ser817Cys) and p.(Met1445Val) variants. &This mutation was identified in an *LRP6* mutant allele with additional p.(Ser127Thr) variant. #This mutation was identified in an *LRP6* mutant allele with additional p.(Ser127Thr) variant from an oligodontia patient who also carried a *WNT10A* p.(Glu167Gln) mutation. §Subjects of this mutation were excluded from statistical analysis due to unconfirmed dental phenotype.

References of supplementary materials

1. Prasad, M.K.; Geoffroy, V.; Vicaire, S.; Jost, B.; Dumas, M.; Le Gras, S.; Switala, M.; Gasse, B.; Laugel-Haushalter, V.; Paschaki, M.; et al. A targeted next-generation sequencing assay for the molecular diagnosis of genetic disorders with orodental involvement. *J. Med Genet.* **2016**, *53*, 98–110, doi:10.1136/jmedgenet-2015-103302.
2. Massink, M.P.; Créton, M.A.; Spanevello, F.; Fennis, W.M.; Cune, M.S.; Savelberg, S.M.; Nijman, I.J.; Maurice, M.M.; van den Boogaard, M.J.; van Haaften, G. Loss-of-Function Mutations in the WNT Co-receptor LRP6 Cause Autosomal-Dominant Oligodontia. *Am J Hum Genet* **2015**, *97*, 621-626, doi:10.1016/j.ajhg.2015.08.014.
3. Yu, M.; Fan, Z.; Wong, S.W.; Sun, K.; Zhang, L.; Liu, H.; Feng, H.; Liu, Y.; Han, D. Lrp6 Dynamic Expression in Tooth Development and Mutations in Oligodontia. *J Dent Res* **2021**, *100*, 415-422, doi:10.1177/0022034520970459.
4. Ockeloen, C.W.; Khandelwal, K.D.; Dreesen, K.; Ludwig, K.U.; Sullivan, R.; van Rooij, I.; Thonissen, M.; Swinnen, S.; Phan, M.; Conte, F.; et al. Novel mutations in LRP6 highlight the role of WNT signaling in tooth agenesis. *Genet Med* **2016**, *18*, 1158-1162, doi:10.1038/gim.2016.10.
5. Whyte, M.P.; McAlister, W.H.; Zhang, F.; Bijanki, V.N.; Nenninger, A.; Gottesman, G.S.; Lin, E.L.; Huskey, M.; Duan, S.; Dahir, K.; et al. New explanation for autosomal dominant high bone mass: Mutation of low-density lipoprotein receptor-related protein 6. *Bone* **2019**, *127*, 228-243, doi:10.1016/j.bone.2019.05.003.
6. Brance, M.L.; Brun, L.R.; Cóccaro, N.M.; Aravena, A.; Duan, S.; Mumm, S.; Whyte, M.P. High bone mass from mutation of low-density lipoprotein receptor-related protein 6 (LRP6). *Bone* **2020**, *141*, 115550, doi:10.1016/j.bone.2020.115550.
7. Wang, H.; Liu, Y.; Zheng, Y.; Zhao, X.; Lin, S.; Zhang, Q.; Zhang, X. A novel missense mutation of LRP6 identified by whole-exome sequencing in a Chinese family with non-syndromic tooth agenesis. *Orthod Craniofac Res* **2021**, *24*, 233-240, doi:10.1111/ocr.12424.

8. Zhou, M.; Zhang, H.; Camhi, H.; Seymen, F.; Koruyucu, M.; Kasimoglu, Y.; Kim, J.W.; Kim-Berman, H.; Yuson, N.M.R.; Benke, P.J.; et al. Analyses of oligodontia phenotypes and genetic etiologies. *Int J Oral Sci* **2021**, *13*, 32, doi:10.1038/s41368-021-00135-3.
9. Keskin, G.; Karaer, K.; Uçar Gündoğar, Z. Targeted next-generation sequencing (NGS) analysis of mutations in nonsyndromic tooth agenesis candidate genes : Analysis of a Turkish cohort. *J Orofac Orthop* **2021**, doi:10.1007/s00056-021-00284-4.
10. Goto, H.; Kimura, M.; Machida, J.; Ota, A.; Nakashima, M.; Tsuchida, N.; Adachi, J.; Aoki, Y.; Tatematsu, T.; Takahashi, K.; et al. A novel LRP6 variant in a Japanese family with oligodontia. *Hum Genome Var* **2021**, *8*, 30, doi:10.1038/s41439-021-00162-w.
11. Huang, Y.X.; Gao, C.Y.; Zheng, C.Y.; Chen, X.; Yan, Y.S.; Sun, Y.Q.; Dong, X.Y.; Yang, K.; Zhang, D.L. Investigation of a Novel LRP6 Variant Causing Autosomal-Dominant Tooth Agenesis. *Front Genet* **2021**, *12*, 688241, doi:10.3389/fgene.2021.688241.
12. Basha, M.; Demeer, B.; Revencu, N.; Helaers, R.; Theys, S.; Bou Saba, S.; Boute, O.; Devauchelle, B.; Francois, G.; Bayet, B.; et al. Whole exome sequencing identifies mutations in 10% of patients with familial non-syndromic cleft lip and/or palate in genes mutated in well-known syndromes. *J Med Genet* **2018**, *55*, 449-458, doi:10.1136/jmedgenet-2017-105110.
13. Dinckan, N.; Du, R.; Petty, L.E.; Coban-Akdemir, Z.; Jhangiani, S.N.; Paine, I.; Baugh, E.H.; Erdem, A.P.; Kayserili, H.; Doddapaneni, H.; et al. Whole-Exome Sequencing Identifies Novel Variants for Tooth Agenesis. *J Dent Res* **2018**, *97*, 49-59, doi:10.1177/0022034517724149.
